# Risk of prenatal depression and stress treatment: alteration on serotonin system of offspring through exposure to Fluoxetine

**DOI:** 10.1038/srep33822

**Published:** 2016-10-05

**Authors:** Siran Pei, Li Liu, Zhaomin Zhong, Han Wang, Shuo Lin, Jing Shang

**Affiliations:** 1 State Key Laboratory of Natural Medicines, China Pharmaceutical University, Nanjing Jiangsu, 210009, China; 2 Jiangsu Key Laboratory of TCM Evaluation and Translational Research, China Pharmaceutical University, Nanjing, Jiangsu, 210009, China; 3 Department of Molecular, Cell and Developmental Biology, University of California, Los Angeles, CA 90095, USA; 4 Center for Circadian Clocks, School of Biology & Basic Medical Sciences, Medical College, Soochow University, Suzhou Jiangsu 215003, Jiangsu, China; 5 Key Laboratory of Tibetan Medicine Research, Northwest Institute of Plateau Biology, Chinese Academy of Sciences, Xining, Qinghai, 810008, China; 6 Qinghai Key Laboratory of Tibetan Medicine Pharmacology and Safety Evaluation, Northwest Institute of Plateau Biology, Chinese Academy of Sciences, Xining, Qinghai, 810008, China

## Abstract

Fluoxetine is widely used to treat depression, including depression in pregnant and postpartum women. Studies suggest that fluoxetine may have adverse effects on offspring, presumably through its action on various serotonin receptors (HTRs). However, definitive evidence and the underlying mechanisms are largely unavailable. As initial steps towards establishing a human cellular and animal model, we analyzed the expression patterns of several HTRs through the differentiation of human induced pluripotent stem (hiPS) cells into neuronal cells, and analyzed expression pattern in zebrafish embryos. Treatment of zebrafish embryos with fluoxetine significantly blocked the expression of multiple HTRs. Furthermore, fluoxetine gave rise to a change in neuropsychology. Embryos treated with fluoxetine continued to exhibit abnormal behavior upto 12 days post fertilization due to changes in HTRs. These findings support a possible long-term risk of serotonin pathway alteration, possibly resulting from the “placental drug transfer”.

Drugs used during pregnancy can have side effects on a developing fetus if it traverses through the placental barrier^1^. A number of antenatal medication exposures are known to cause birth defects^2^. However, due to ethical issue, fetal risk assessment studies remain extremely limited, especially when investigating the long-term side effects on offsprings. The number of patients suffering from depression has increased markedly overtime, thereby increasing the prescription of antidepressants. Antidepressant drugs vary significantly in effects between individuals and are typically taken over long periods of time. A challenge in the field of antidepressant drug discovery is to understand the effects of antidepressants on a developing fetus.

The World Health Organization predicts that by the year 2020, depression will be the second largest contributor to global disability in men and women of all ages^3^. This rising prevalence of mental illness might be contributed by certain adverse influence from adolescence^4^, due to a very early exposure of anti-depression drugs on a fetus when mothers are on medication. It is estimated that there is a 10–16% chance of depression during pregnancy^5^ and 25% of depressed women continue their antidepressant therapy during gestation^6^.

Fluoxetine (Flu) belongs to a class of antidepressants called selective serotonin re-uptake inhibitors (SSRIs). SSRIs work by inhibiting the reuptake of 5-hydroxytryptamine (5-HT, serotonin) and maintaining serotonin levels within the synaptic cleft. As one of the top 10 most common medication for depression, Flu has achieved much better success than others due to its proven safety in adults^6^. Therefore, SSRIs are also the drugs of choice for treating depressed pregnant and postpartum women^7^. In these cases, SSRIs can reach the fetus via the placenta or to a child through breast-feeding^8^. This was shown through detection of SSRIs in the brain of the children during development^9^.

Several epidemiology studies have suggested that the use of SSRIs during pregnancy, a critical period for offspring’s neural development, is associated with disorders relating to attention, emotion, and arousability^10^,^11^. These anxiety- or depression-like behavior symptoms appear to be in conflict with the beneficial effect of SSRI in adults and are hence termed ‘paradoxical’^10^,^12^,^13^,^14^,^15^. With the rise in depression rate, we speculate that the number of pregnant individuals using SSRIs would continue to rise. To address the issue of fetal long-term exposure, we need to better understand how SSRIs can affect neuronal development.

As one of the earliest neurons to arise, serotonergic neurons can release serotonin through growing axons before conventional synapses are established^16^. Therefore, the serotonin signals through multiple receptors exert their biological functions very early during development. Moreover, the bindings modulate a number of developmental events, including cell division, neuronal migration, cell differentiation and synaptogenesis^18^,^19^,^20^.

The superfamily of vertebrate serotonin receptors (HTRs) is divided into seven different families that consist of fourteen different receptors^21^. For example, the largest class of HTRs is the HTR1 family, which is characterized by an intronless coding sequence and includes five subtypes: A, B, D, E, and F^22^. HTRs play such important roles in the function, regulation, and development of the neural system. Within the central nervous system, HTR1A is expressed in many areas. The activities of HTR1A functions were able to relieve anxiety and depression^23^. Interestingly, HTR2A in adult appears to have opposite effects^24^. Little is known about HTR5A function in the CNS, but the expression of HTR5A in brain areas implicates a role in learning and memory formation^25^. However, neither normal expression patterns of the different HTRs during the early neural development nor the aberrant alternation caused by SSRIs have been thoroughly studied in any species. The use of human fetus *in vivo* for earlier study raises many ethical concerns. Here we establish two models to study the genes’ expression patterns and the affects of Flu treatment during early development. The use of these models overcomes the ethical burden of using human embryonic stem cells and fetal samples. Furthermore, it offers a more practical approach.

We used human induced pluripotent stem (hiPS) cells in our study to obtain both CNS neurons and neural crest cells (NCs), which are capable of differentiating into PNS neurons^26^,^27^. We then analyzed expression of HTRs during neuronal differentiation of the hiPS cells. To gain information of HTR expression patterns and regulation by SSRIs *in vivo*, we utilized zebrafish embryos due to the practicality in small molecule treatment^28^,^29^. In zebrafish, the sequence and genomic organization of HTR genes are similar to the mammalian HTRs^30^. In this study, we analyzed embryonic expression patterns of five HTRs by whole mount RNA *in situ* hybridization and their alteration in response to fluoxetine treatment. We showed some similarity of neuronal expression of HTRs between the hiPS system and zebrafish embryonic development. Most importantly, we detected depression-like symptom in the larval fish treated with fluoxetine at early embryonic stages.

## Results

### Neural induction and differentiation from human induced pluripotent stem cells

**Figure 1:**
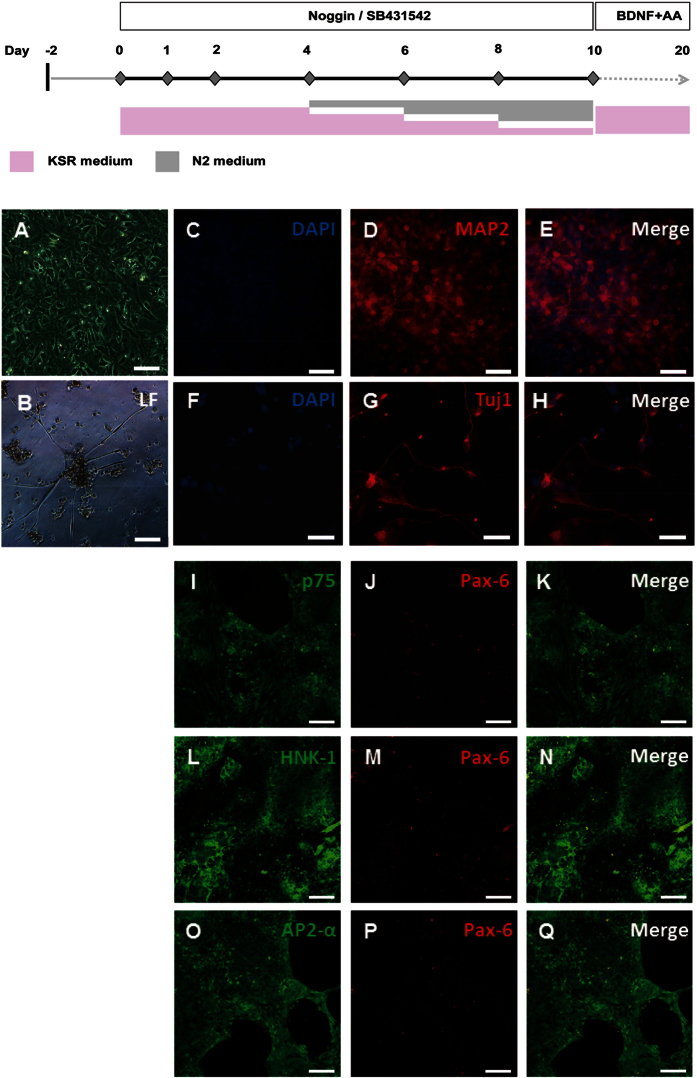
Dual SMAD inhibition allows for a highly efficient neural induction of neuron and neural crest (NC) cells from hiPS cells. (**A**) Differentiation scheme used for neural induction can be achieved with the combination of Noggin, a BMP inhibitor and SB431542, an ALK inhibitor; (**B**) By day 10, the cells appear dramatically thicker with densely packed nuclei and phase-bright ridges; (**C**) By day 20 the typical cell morphology of neuron under light field (LF); (**D–I**) By day 20, the neuron-like cells showed positive for mature neuron marker MAP2 (red) and Tuj1 (red) by immunocytochemistry; (**J–R**) By day 10, the hiPS cells could be selectively directed toward neural crest cells if neural induction began at lower densities. In this case more cells are observed expressing NC markers, p75 (red), HNK-1 (red) and AP-2α (red), but without expressing Pax6 (green), an early marker of neurectodermal differentiation. Nuclei were stained with DAPI (blue). Bars = 200 mm (**B,D–F, J–R**), 400 mm (**C,G–I**).

The initial neural induction from hIPS cells was observed on day 10 of differentiation as previously reported^26^. (Fig. 1) At this time, immunofluorescence staining showed that the NC cells were positive for p75 (Fig. 1I,K), HNK-1 (Fig. 1L,N), AP-2a (Fig. 1O,Q) but negative for Pax-6 (Fig. 1J,M,P), confirming the differentiation of NC cells to those previously described^26^,^31^. An additional 10 days of differentiation under defined factors led to typical morphology of soma and neurites (Fig. 1B), and expression of mature neuronal markers, such as MAP2 (Fig. 1C,E) and Tuj1 (Fig. 1F,H,I). These findings suggested that hIPS cells differentiated into expected neurons.

### Expression of HTRs during neuronal differentiation of hiPS cells

**Figure 2:**
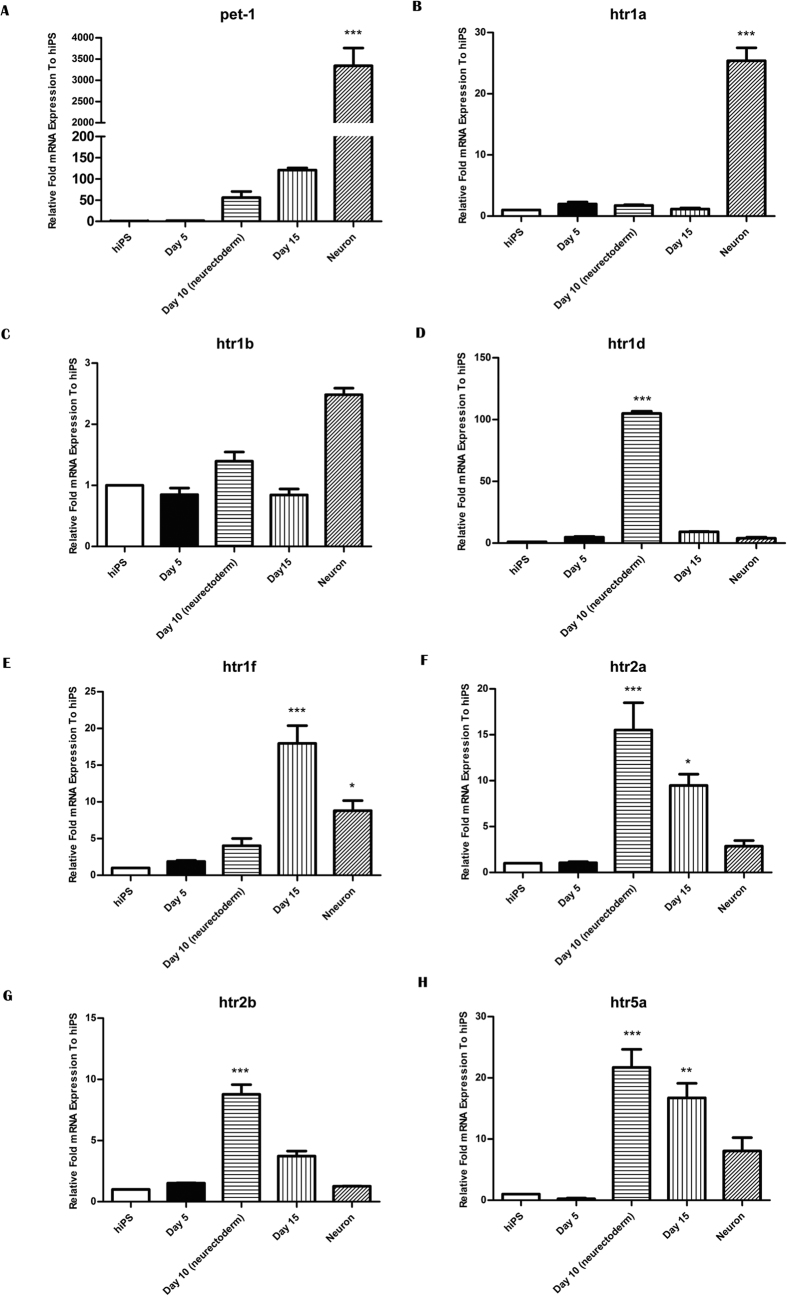
Expression patterns of serotonin receptors from hiPS cells to CNS neurons. Normalized expression tested for *pet-1* (**A**), *htr1a* (**B**), *htr1b* (**C**), *htr1d* (**D**), *htr1f* (**E**), *htr2a* (**F**), *htr2b* (**G**) and *htr5a* (**H**) by qPCR. ***p < 0.005, **p < 0.01, *p < 0.05, one-way analysis of variance (ANOVA) with Tukey’s post-hoc test was used between the hiPS cells group and each time point follow. All error bars show mean ± s.e.m.

**Figure 3:**
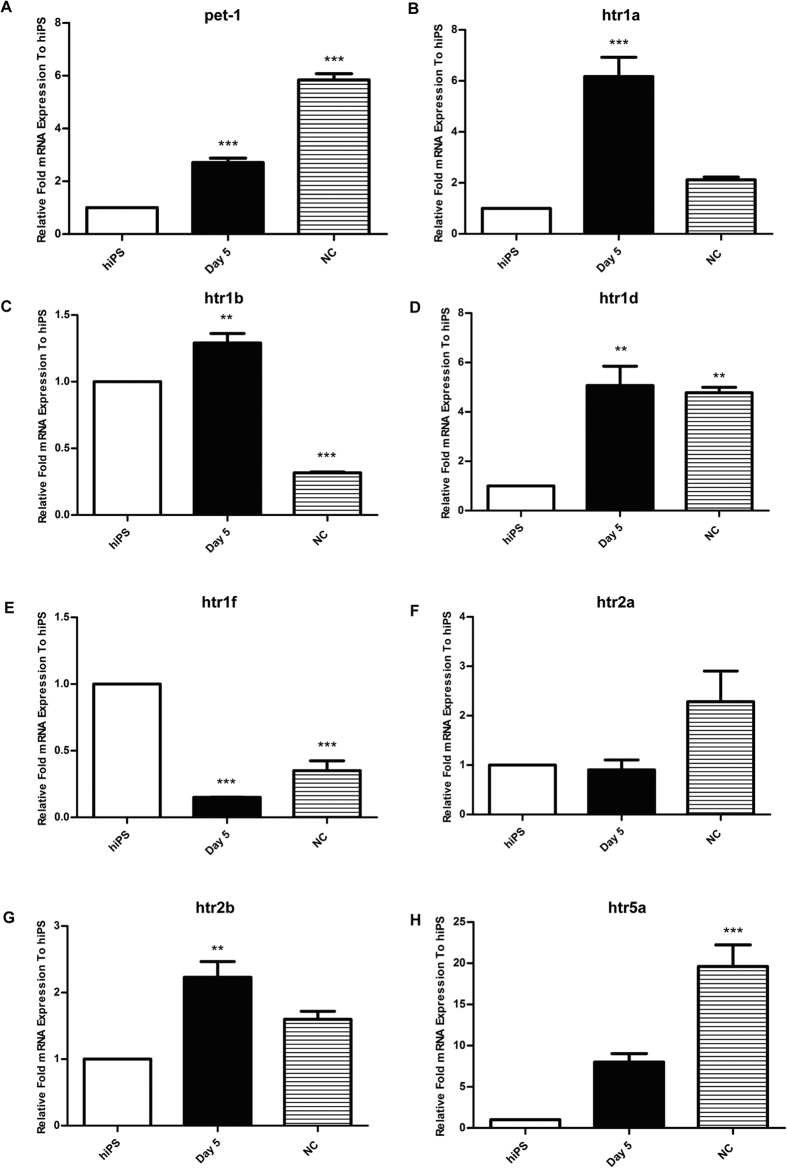
Expression patterns of serotonin receptors from hiPS cells to NC cells. Normalized expression tested for *pet-1* (**A**), *htr1a* (**B**), *htr1b* (**C**), *htr1d* (**D**), *htr1f* (**E**), *htr2a* (**F**), *htr2b* (**G**) and *htr5a* (**H**) by qPCR. ***p < 0.005, **p < 0.01, one-way ANOVA with Tukey’s post-hoc test was used between the hiPS cells group and each time point follow. All error bars show mean ± s.e.m.

**Figure 4:**
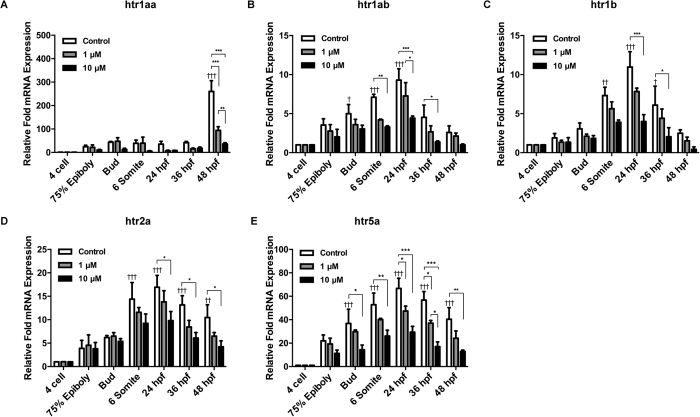
Quantitative changes of the expression of serotonin receptors during the early development of zebrafish embryos under fluoxetine administration. Real-time PCR was performed to confirm the relative expression level changes of *htr1aa* (**A**), *htr1ab* (**B**), *htr1b* (**C**), *htr2a* (**D**) and *htr5a* (**E**) with or without the treatment of fluoxetine in two doses. Two-way ANOVA with Tukey’s multiple comparisons test was used for analysis; n ≥ 20 per group, ***p < 0.005, **p < 0.01, *p < 0.05, manes the statistically significance between different doses in each of six time points, ^†††^*p* < 0.005, ^††^*p* < 0.01, ^†^*p* < 0.05 manes the statistically significance between different time points of control groups. All error bars show mean ± s.e.m.

During the differentiation period, several time points were selected to quantify the mRNA expression of *pet-1*, *htr1a*, *htr1b*, *htr1d*, *htr1f*, *htr2a*, *htr2b* and *htr5a* through qPCR (Figs 2 and 3A–H). The mRNAs expression of undifferentiated hiPS cells was considered as control. *Pet-1* expression gradually increased during CNS neuron differentiation. At the neuroectodermal and neuronal time points, *Pet-1* expression was 50 folds and 3000 folds higher than in hiPS cells, respectively (Fig. 2A), while increases in *Pet-1* was not as significant as in the CNS direction during the NC differentiation (Fig. 3A), suggesting that there was a different expression pattern of *Pet-1* between CNS and NC cells. The mRNA expression of *htr1a* remained steady before the neuron time point, and increased more than 20 folds at its final point. However, in the NC direction there was a significant increase by day 5, though it was followed by an immediate decrease without significant change compared with control in the end. This pattern was different from that of the neuroectodermal stage probably because *htr1a* is the main auto-receptors on serotonin neurons. Therefore the neuroectodermal pattern of *htr1a* is similar to *Pet-1* (Fig. 3A,B). While in the NC direction, the data showed a reverse change of *htr1a* (Fig. 4B). This could be explained by the fact that the induction of NC depends on serotonin signal to regulate cells’ fate to NC through *htr1a*. The mRNA expression of *htr1b* and *htr1a* did not change significantly before differentiation to neurons but *htr1a* increased more than 20 folds and htr1b two folds compared to control at the final stage of neuronal differentiation (Fig. 2B,C). The mRNA expression of *htr1d*, *1f*, *2a*, *2b*, *5a* reached the peak from 10 to 100 folds at the neuroectodermal time point. During the second neuronal differentiation period, their expression gradually decreased and was not significant different from control at the end, except *htr1f* (Fig. 2D–H).

During NC differentiation, the *htr1a* and *htr1b* expression displayed an initial increase followed by a decrease (Fig. 3B,C). The *htr1d* mRNA expression significant increased and maintained a high level of expression (Fig. 3D), while the *htr1f* expression decreased and stayed at a lower level compared to control (Fig. 4E). No significant change of *htr2a* expression was observed (Fig. 3F). Meanwhile, *htr2b* (Fig. 3G) expression had a rising trend at first and then dropped. Finally, *htr5a* increased significantly and reached more than 20 folds compared to the control (Fig. 3H).

### Expression of HTRs in zebrafish during embryonic development

We analyzed expression of zebrafish htrs by qPCR at the 4-cell stage, 75% epiboly, tail bud stages, 6-somite stage, 24 hpf, 36 hpf and 48 hpf (Fig. 4). The results showed that the expression of *htr1aa* was relatively lower than other htrs at the time point of 4-cell, but it elevated at 75% epiboly stage significantly and remained a high level till 36 hpf stage, then increased sharply to more than 200 fold at 48 hpf comparing to 4-cell stage (Fig. 4A). Though the variation trend of *htr1ab, 1b, 2a, 5a* had similar trends, which started from 4-cell stage, peaking at 24 hpf, and then gradually reducing, the expression patterns of *htr2a* and *htr5a* have more significant increase and stays at a higher level than *htr1ab and htr 1b*. (Fig. 4B–E).

**Figure 5:**
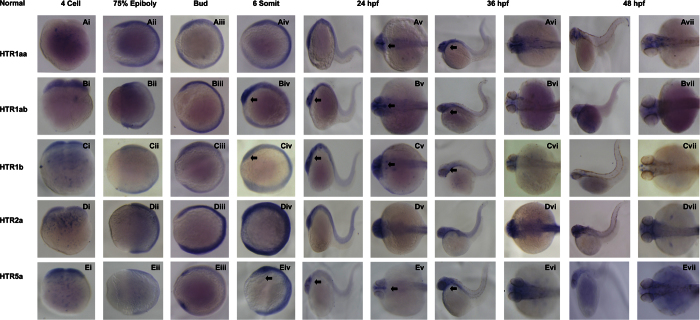
Normal Expression patterns of serotonin receptors during early development of zebrafish embryos. Expression of *htr1aa* (A), *htr1ab* (B), *htr1b* (C), *htr2a* (D) and *htr5a* (E) on 7 developmental phases of zebrafish early development: 4-cell, 75% Epiboly, Bud, 6 Somite, 24 hpf, 36 hpf and 48 hpf (i–vii) in both lateral and dorsal whole mount views. Embryos were positioned anterior left and viewed laterally.

To gain information on the spatial patterns of HTR expression, whole mount RNA *in situ* hybridization was used (Fig. 5). The results showed that *htrs are* express as early as in the 4-cell stage (Fig. 5Ai–Ei). *Htrs* expression is detected ubiquitously throughout the embryo at 75% epiboly and tail bud stage (Fig. 5Aii–Eii,Aiii–Eiii). Among those analysed, *htr1ab*, *htr1b* and *htr5a* showed stronger signal in the poles of the development embryo. This expression pattern was more significant at 6-somite stage (Fig. 5Biv,Civ,Eiv, black arrow). At 24 hpf, *htr1ab*, *htr1b* and *htr5a* were expressed specifically in the brain (Fig. 5Bv,Cv,Ev, black arrow). Expression of *htr1aa* appeared more robustly in CNS and PNS (Fig. 5 Av, black arrow). Dorsal and lateral views showed *htrs (*except for *htr2a)* were mostly restricted to the neural system from 24 hpf (Fig. 5Av–Ev). *Htr1aa* was expressed in CNS including optic tectum (TeO), anterior pharynx (PH), inferior raphe nucleus (IR), pretectal diencephalic cluster (PP), superior raphe nucleus (SR) and PNS, the area beyond brain (Fig. 5Av). Surprisingly, *htr1ab* displayed a different pattern of expression, mainly in the CNS, including the optic tectum (TeO), anterior pharynx (PH), inferior raphe nucleus (IR), retina and medulla oblongata (MO) and medulla spinallis (MS) (Fig. 5Bv). *htr1b* showed similar expression to *htr1ab* and was observed in the optic tectum (TeO), inferior raphe nucleus (IR), retina and medulla oblongata (MO), medulla spinallis (MS) and intermediate reticular formation (IMRF) (Fig. 5Cv). *htr5a* showed the highest level of tissue specificity, with expression in the inferior raphe nucleus (IR) and optic tectum (TeO) (Fig. 5Ev). Unlike the other htrs, the signal of *htr2a* showed a ubiquitous expression pattern (Fig. 5Dv). With the development process, the expression of HTRs was decreased at 24 hpf. Moreover, there was a general tendency that more and more *htrs* clustered to brain (Fig. 5Avi–Evi). At 48 hpf, the expression of *htr1b* and *htr5a* was much lower (Fig. 5Cvii,Evii), showing dispersed expression in the nervous system.

### Fluoxetine induced change of HTR expression in zebrafish embryos

Fluoxetine was directly added to fish water to study its effect on HTRs during zebrafish development. Levels of HTR expression were determined using qPCR. Fish water containing fluoxetine had an inhibitory effect on the expression of HTRs in a dose-dependent manner (Fig. 4). For HTR1aa, doses above 10 uM almost completely and continuously suppressed its expression from as early as 4-cell stage, while doses lower than 1 uM was only effective later from 24 to 48 hpf, (Fig. 4A) For other HTRs, fluoxetine treatment also suppressed expression at all stages in a dose dependent manner (Fig. 4B–E).

**Figure 6:**
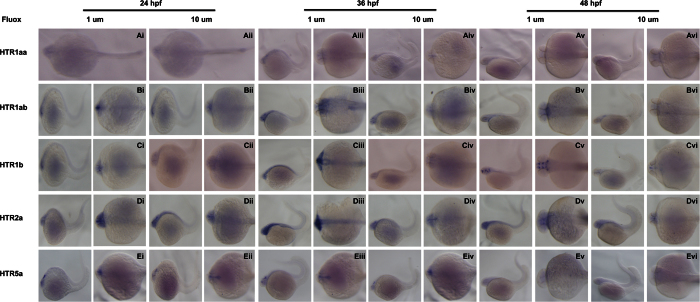
Expression patterns of serotonin receptors during the early development of zebrafish embryos under fluoxetine administration. Expression of *htr1aa* (A), *htr1ab* (B), *htr1b* (C), *htr2a* (D) and *htr5a* (E) on 3 developmental phases of zebrafish early development: 24 hpf, 36 hpf and 48 hpf (i–iii) in both lateral and dorsal whole mount views under 1 μM and 10 μM fluoxetine administration. Embryos were positioned anterior left and viewed laterally.

Next, effect of fluoxetine on expression of HTRs *in vivo* was investigated through RNA *in situ* hybridization. The photos were taken after Flu treatment from 1000 cell-stage to 24 hpf. As expected, fluoxetine treatment inhibited expression of all the *htrs* at the tested time points and in a dose-dependent manner, consistent with qPCR results. The most notable expression of HTRs in neurons was significantly inhibited. For *htr1aa*, its neuronal expression became undetectable after 1 uM treatment (Fig. 6Ai–Av). Expression of *htr1ab*, *htr1b* and *htr5a* was less sensitive to fluoxetine but still showed a dose-dependent inhibition (Fig. 6Bi–Bv,Ci–Cv,Ei–Ev). *Htr2a* was not inhibited by fluoxetine (Fig. 6Di-Dv).

### Fluoxetine treatment evoked hypolocomotor effect on zebrafish larvae

**Figure 7:**
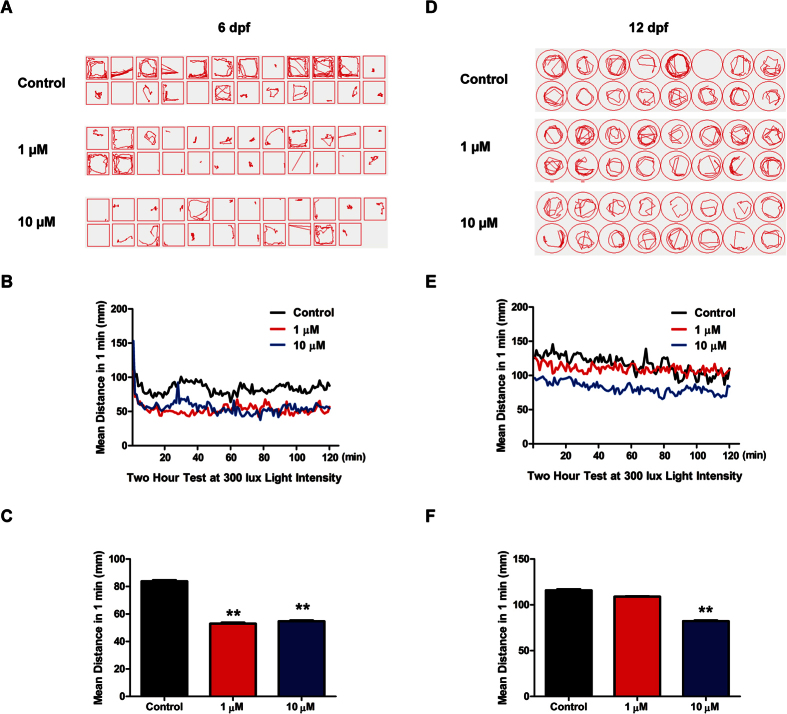
The hypolocomotor behavior of zebrafish larva induced by fluoxetine. (**A,D**) Recorded swimming tracks of 3 groups zebrafish. Each red line represents the swimming track of 1 minute recording randomly selected from the 2-hour experiment on both 6 dpf and 12 dpf. (**B,E**) The locomotor activities of each minute for all zebrafish larva in 3 groups on 6 dpf and 12 dpf. (**C,F**) The statistic analysis of the average locomotor activities, n ≥ 20 per group, ***p < 0.005, **p < 0.01, *p < 0.05, one-way ANOVA with Tukey’s post-hoc test was used between the control group and each dose fluoxetine group. All error bars show mean ± s.e.m.

After analysis of HTR expression, the effects of Flu were analyzed on the behavior of the zebrafish. On 6 dpf, with 3 days of fluoxetine treatment, locomotor activity was analyzed to investigate the change in zebrafish larva behavior. On 6 dpf, both 1 uM and 10 uM fluoxetine treatment groups had fewer tracks compared to control group (Fig. 7A). A measurement was taken every minute for 2 hrs and the treatment groups consistently showed lower activity compared to control group (Fig. 7B). The difference of average activity between the treated and control zebrafish was statistically significant (Fig. 7C). When this same test was run on 12 dpf, 1 uM fluoxetine treatment no longer had any significant hypolocomotor effect. However, the higher dose (10 uM) still significantly inhibited the zebrafish locomotor activity (Fig. 7D–F).

## Discussion

In our work, we used both hiPS cells and zebrafish embryos to analyze expression of serotonergic system. We found that expression pattern of HTRs during hiPS cells’ differentiation *in vitro* and embryonic development of zebrafish *in vivo* showed similar trends. As previously studies, *pet-1* factor was revealed as a precise marker of developing and adult serotonin neurons^32^. Also it functions specifically in the differentiation and maintenance of these neurons^33^, which was used here for serotonin neuron identity during hiPS cells differentiation. During neuronal induction of hiPS cells, *pet-1* was significantly increased, suggesting that the serotonin neurons are emerging in the very early stages, secreting serotonin and binding to its receptors. Comparing CNS and PNS, *pet-1* expression was much stronger in CNS providing evidence that the mature serotonin neurons were included. The *pet-1* positive cells in CNS are most likely expressing *htr1a* since *pet-1* and *htr1a* have a very similar induction pattern. It is therefore conceivable that formation of serotonin neurons is also induced very early during CNS development in humans. As mentioned above, women use SSRIs during pregnancy for treating depression^7^. This could have an immediate impact on the development of serotonin system through regulating the expression of serotonin receptors.

Consistent with the finding that serotonin receptor *htr1a* expression is prominent in CNS cells induced from hiPS cells, we find that *htr1aa* is highly expressed in the nervous system of zebrafish during the period of CNS development *in vivo*. The zebrafish genome contains *htr1aa* and *htr1ab* but we believe that htr1aa is the ortholog of mammalian *htr1a* due to the observation that *htr1ab* has an expression pattern more similar to that of htr1b (Figs 4 and 5), although *htr1ab* shares sequence homology with both *htr1aa* and *htr1b*. Interestingly, *htr1aa* expression is the most sensitive to fluoxetine treatment, a representative SSRI that is most widely used by pregnant women. Through a dose response analysis, we observed that 1 μM and 10 μM are appropriate for our studies on zebrafish embryos due to the lack of adverse effects on morphology. Furthermore, these doses are compatible to doses exposed by human fetuses. Both of the 1 uM and 10 uM doses showed the significant changes of the serotonin system. During zebrafish embryogenesis, fluoxetine clearly has a profound negative impact on expression of htr1aa and other HTRs as revealed through qPCR and *in situ* hybridization analysis. Our study represents the first to characterize expression patterns of multiple HTRs in zebrafish and establish that fluoxetine can directly inhibit HTR expression. The first two days of external development of zebrafish embryos should reflect the early human development before birth, at which neurogenesis is established. If reduction of HTRs, at this developmental stage, has a long-term effect on the individual’s ability to respond to serotonin signaling, our findings would imply that there is a risk of causing depression later in life due to “placental drug transfer” of SSRIs. Supporting this hypothesis is that fluoxetine treatment during the early development indeed effectively induced behavior deficiency of hypolocomotor in the larval stages. Hypolocomotor activity has been used as a key symptom of depression in zebrafish. After removing fluoxetine, zebrafish larvae still showed hypolocomotor behavior, suggesting there is a potential long-term effect of fluoxetine treatment.

**Figure 8:**
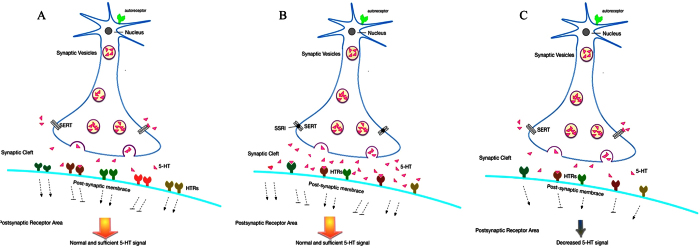
Potential mechanism offspring depression induced by SSRIs. (**A**) The natural level of both serotonin quantity and HTRs expression ensure the normal serotonin signal. (**B**) During embryo early development, to maintain the regular serotonin signal, fewer HTRs express reply more available serotonin resulting from SSRIs that are taken by depressed pregnant. (**C**) Lower serotonin signal appears after the stop using of SSRIs as the fewer HTRs expression.

Our findings that HTRs are inhibited by fluoxetine in zebrafish embryos may offer potential mechanism underlying the ‘paradoxical’ phenomena mentioned above. Alternation of HTRs induced by SSRIs may have long-term impact on behavior of offspring through direct and indirect mechanisms. Directly, SSRIs work through inhibiting the reuptake of the serotonin located in the synaptic cleft (Fig. 8A). When pregnant women with severe depression take SSRIs, the drugs bring the concentration of serotonin back to a normal level in the mother but this action might sprout serotonin above normal level in the developing fetus. As a feedback mechanism, overabundance of serotonin in the baby reduces HTRs expression in a feedback manner (Fig. 8B). If this reduced level of HTRs becomes a default setting and persists after birth, these babies would face insufficiency of serotonin signaling due to insufficiency of HTRs later in life (Fig. 8C). Consequently, ‘paradoxical’ anxiety- or depression-like behavior symptoms are developed in the offspring of mothers who took SSRIs during pregnancy.

Indirectly, before serotonin becomes a neurotransmitter, it acts as a developmental signal regulating cell growth and differentiation of the nerve system. Thus, early alterations in serotonergic signal may influence other developmental processes, such as increased neonatal serum corticosteroid binding globulin levels at birth^34^, attenuated basal salivary cortisol levels^35^, and decreased serum corticosterone response to stress as shown in prenatally stressed mouse offspring^36^. Likewise, the use of SSRI medications by pregnant mothers and young children may pose risks of emotional disorders later in life^13^.

## Materials and Methods

### hiPS Cells Derivation, Culture and Neural Differentiation

We derived hiPS cells from human fibroblasts (ATCC, CCD-1079sk) following previous protocols^37^,^38^ (Supplementary material). The differentiation procedure is shown in Fig. 1. Clones of hiPS were disaggregated using accutase (Sigma, A6964) to single hiPS cells. After washing and centrifuging cells to remove accutase, single hiPS cells were plated on matrigel coated dishes, which were pre-warmed for 1 hour at 37 °C, at a density of 20,000–50,000 cells/cm^2^ (for CNS neuron) or 10,000 cells/cm^2^ (for NC cells) in hiPS cells medium containing ROCK-inhibitor Y-27632 (Tocris, 1254). The ROCK inhibitor was withdrawn, and hiPS cells were changed to conditional medium (CM, hiPS cells medium after culturing with MEF for 24 h) until cells reach the appropriate density. The initial differentiation medium conditions included knockout serum replacement (KSR) containing two kinds of inhibitors, 200 ng/mL of Noggin (R&D, 6057-NG) and 10 μM SB431542 (Tocris, 1614). During neural induction, the KSR medium was gradually withdrawn (100%, 75%, 50%, 25%) with increasing amounts of N2 medium, which contained N2 supplement (Invitrogen, 17502048) and the same inhibitors.

All animal experiments were performed in accordance with the guidelines established by China Pharmaceutical University Animal Ethics Committee. All animal experimental protocols were approved by China Pharmaceutical University Animal Ethics Committee.

### Zebrafish maintenance

Wild-type AB line was maintained in a circulating aquaculture system according to standards described in The Zebrafish Book^39^. Embryos were incubated at 28.5 °C and staged according to the description by Kimmel *et al*.^40^. Fluoxetine (Sigma-Aldrich, F132) was dissolved in Milli Q water to make stock solutions and then diluted with fresh fish water to final treatment concentration. PTU (Sigma-Aldrich, P7962) was dissolved in water to make stock solutions and then diluted with fresh fish water to 0.0003% for all treatments.

Zebrafish protocol and maintenance were performed using methods approved by the University of California, Los Angeles Institutional Animal Care and Use Committee and Soochow University Animal Use and Care Committee.

### Immunocytochemistry

Cells were fixed with 4% paraformaldehyde for 30 min. Nonspecific sites were blocked with 1% BSA/PBS (for extracellular epitopes) or 1% BSA/PBS/0.5% triton X-100 (for intracellular epitopes) for 30 min at 37 °C. Then the specimens were incubated with primary antibodies at 4 °C overnight. Excess primary antibodies were removed by washing with PBS for 5 min and repeated 3 times. Secondary antibodies labeled with TRITC/FITC were used and incubated with samples at room temperature for 1 hr. The excess secondary antibodies were removed by washing with PBS for 5 min and repeated 3 times in the dark. Primary antibodies used included Oct4, Sox2, Nanog, SSEA4, Tra-1-60 and Tra-1-81 (Sidansai, A-P1-1KT), MAP2 (Abcam, cat. no. ab11267), TUJ1(Covance, cat. no. MMS-435P), p75 antibody (Advanced targeting systems, cat. no. AB-N07), Pax-6 antibody (Covance, cat. no. PRB-278P), AP2 antibody (DSHB, cat. no. 3B5) HNK1 antibody (Sigma, cat. no. C6680).

### RNA Extraction and PCR

RNA was extracted using the Trizol reagent (Invitrogen, 15596018), and RT-PCRs were performed in a two-step way with RT-PCR kit (Takara, DRR036A). PCR was performed with ExTaq (Takara, Japan). Quantitative real-time PCR was performed with Platinum SYBR Green qPCR Supermix UDG (Takara, Japan) and analyzed with the IQ5 real-time PCR system (Bio-Rad).

### RNA *In Situ* Hybridization

Whole mount ISH was performed as described previously^41^. PCR fragments of *htr1aa, htr1ab, htr1b, htr2a* and *htr5a* were confirmed by sequences and used as templates for *in vitro* transcription. The stages of the embryos were chosen as follows: 4-Cell stage, Bud stage, 75% Epiboly stage, 6-Somite stage, 24 hpf, 36 hpf (1.5 dpf) and 48 hpf (2 dpf). Embryos older than 24 hpf were treated with 0.0003% phenylthiourea (PTU) to prevent the development of pigments. Embryos were fixed by 4% paraformaldehyde solution at 4 °C overnight or stored at −20 °C in methanol. Prior to staining, samples were rehydrated using PBST, followed by proteinase K treatment on embryos older than 24 hpf. All probes used for hybridization were developed by anti-digoxingenin-AP antibody (Roche) and BCIP/NBT color development substrate (Promega). Photographs were taken by AxioCam MRc5 CCD sensor and AxioVision AC software (Zeiss, Germany).

### Locomotor Activities

The zebrafish embryos were divided into three groups (control, fluoxetine 1 um and fluoxetine 10 um) and each group contained 24 embryos. Fluoxetine was treated from 1000-cell stage to 72 hpf. All the embryos were then transferred into normal fish water and allowed to develop into larval stages. Locomotor activity was analyzed as described previously with some modifications^42^. These larvae were individually placed in each well of a 96-well plate (for 6 day old larvae) or 48-well plate (for 12 day old larvae). Locomotor activities of each larva were recorded for 2 hours under 300 lux light conditions using an automated video-tracking system (Videotrack, ViewPoint Life Sciences) and analyzed using Zebralab3.10 software (ViewPoint Life Sciences). The instrument was placed in a chamber with a constant temperature of 25 °C.

### Statistical analysis

All data are presented as mean ± s.e.m. (error bars). Statistical significance was set a priori at 0.05 (p-value ≤ 0.05). For most comparisons between expressions of each time points we used a one-way ANOVA. To compare changes between control and two doses fluoxetine treatment in every single time point, we used a two-way ANOVA. The f-values are calculated and compared to the F-distribution table. Both F-value and p-value tables show in Supplementary material.

## Additional Information

**How to cite this article**: Pei, S. *et al*. Risk of prenatal depression and stress treatment: alteration on serotonin system of offspring through exposure to Fluoxetine. *Sci. Rep.* **6**, 33822; doi: 10.1038/srep33822 (2016).

## Supplementary Material

Supplementary Information
