# Erratum: Risk of prenatal depression and stress treatment: alteration on serotonin system of offspring through exposure to Fluoxetine

**DOI:** 10.1038/srep41344

**Published:** 2017-02-13

**Authors:** Siran Pei, Li Liu, Zhaomin Zhong, Han Wang, Shuo Lin, Jing Shang

Scientific Reports 6: Article number: 3382210.1038/srep33822; published online: 10 05 2016; updated: 02 13 2017

In the original version of this Article, additional affiliations for Jing Shang were omitted. The correct affiliations for this Author are listed below:

State Key Laboratory of Natural Medicines, China Pharmaceutical University, Nanjing, Jiangsu, 210009, China.

Jiangsu Key Laboratory of TCM Evaluation and Translational Research, China Pharmaceutical University, Nanjing, Jiangsu, 210009, China.

Key Laboratory of Tibetan Medicine Research, Northwest Institute of Plateau Biology, Chinese Academy of Sciences, Xining, Qinghai, 810008, China.

Qinghai Key Laboratory of Tibetan Medicine Pharmacology and Safety Evaluation, Northwest Institute of Plateau Biology, Chinese Academy of Sciences, Xining, Qinghai, 810008, China.

This error has now been corrected in the PDF and HTML versions of the Article.

