# Supplementary material for: Risk of prenatal depression and stress treatment: alteration on serotonin system of offspring through exposure to Fluoxetine
Source: Sci Rep. 2016 Oct 5;6:33822. doi: 10.1038/srep33822 (PMC5050550; doi:10.1038/srep33822)
Supplement: Supplementary Information [file srep33822-s1.docx]

**Risk of prenatal depression and stress treatment: alteration on serotonin system of offspring through exposure to the Fluoxetine**

Siran Pei^1, 2, 4^, Li Liu^1, 2^, Zhaomin Zhong^3^, Han Wang^3^, Shuo Lin ^4^ *, Jing Shang^1.2^*

^1^State Key Laboratory of Natural Medicines, China Pharmaceutical University, Nanjing 210009, China

^2^Center for Drug Screening, China Pharmaceutical University, Nanjing 210009, China

^3^Center for Circadian Clocks, School of Biology & Basic Medical Sciences, Medical College, Soochow University, Suzhou 215003, Jiangsu, China

^4^Department of Molecular, Cell and Developmental Biology, University of California, Los Angeles, CA 90095, USA

*Author for correspondence (shuolin@ucla.edu, [shangjing21cn@163.com](mailto:shangjing21cn@163.com))

Key words: Serotonin; Selective serotonin re-uptake inhibitors (SSRIs); induced pluripotent stem cells; zebrafish; expression pattern

**Supplementary material**

hiPS Cell Derivation and Culture

The human genes encoding the transcription factors Oct4, Nanog, Sox2, Lin28, c-myc and Klf4 (Sidansai, cat. no. LV01-LV06) were subcloned into self-inactivating lentiviral vectors driven by the human phosphoglycerate kinase (PGK) promoter. Lentiviral vector supernatants were produced by triple co-transfection of the plasmid DNA encoding the vector, pCMVΔR8.91 and pUCMD.G into 293T cells. Human fibroblasts (ATCC, CCD-1079sk) were seeded at 1.5×10^4^ cells/cm^2^ in Eagle's Minimum Essential Medium supplemented with 10% fetal bovine serum (FBS). The following day the fibroblasts were transduced with equal amounts of supernatants of the six lentiviral vectors in the presence of 4 ug/ml polybrene for 16h. Six days after transduction, fibroblasts were harvested by trypsinization and plated at 2×10^4^ cells per 60 mm^2^ dish on a feeder layer of Co_60_-treated mouse embryonic fibroblasts (MEF-CF-1, Sidansai). The next day, the medium was switched to hESC medium. Colonies with a human ES cell-like morphology (iPS cell colonies) first became visible 12 days after transduction with 6 factors.

hiPS cells were cultured and expanded by growth on a layer of irradiated MEF feeder layer using human embryonic stem (hES) cell medium containing Dulbecco’s Modified Eagle Medium with F12 (DMEM/F12, 1:1 ratio, Invitrogen, 11330) supplemented with 20% Knockout Serum Replacement (Invitrogen, 10828), 100X MEM Non-Essential Amino Acids Solution (Invitrogen, 11140), 200mM L-Glutamine solution (Invitrogen, 25030), 0.1 mM β-mercaptoethanol (Sigma, M7522), and 4ng/mL basic fibroblast growth factor (bFGF, Invitrogen, 13256-029) in Matrigel (BD,356234) pro-coated flask. Colonies were observed on a daily basis, and any colonies exhibiting spontaneous differentiation were removed. hiPS medium was replaced on a daily basis. iPS cells were passed every 7 days onto new irradiated MEFs using a 0.1 mg/mL collagenase IV (Invitrogen, 17104019) in DMEM/F12.

The generation of human induced pluripotent stem cells from HF.

We generated iPS Cells from adult human newborn foreskin fibroblasts (S1.A) following the previous protocol ^[1,2]^. hiPS cell lines were confirmed positive for the qualification tests (S1). The hiPS cells are similar to the human embryonic stem (ES) clone tended to form packed clones with a high nucleus/cytoplasm ratio (S1. B) and displayed a high level of alkaline phosphatase (S1.C). To make sure of the undifferentiated state of the cells, pluripotency-associated genes were detected with immunofluorescence before the NSC differentiation. The results showed that those cells expressed high levels of pluripotency-associated endogenous proteins, Nanog, SSEA3, SSEA4, Tra-1-60 and Tra-1-81 (S1. D-H). Teratoma formation through injecting human iPS cells subcutaneously into dorsal flanks of immunodeficient (SCID) mice was used to test the pluripotency *in vivo*. Histological examination showed that the tumor transferred to various tissues, including ectoderm (S1.I), mesoderm (S1.J) and endoderm (S1.K) tissues.

**
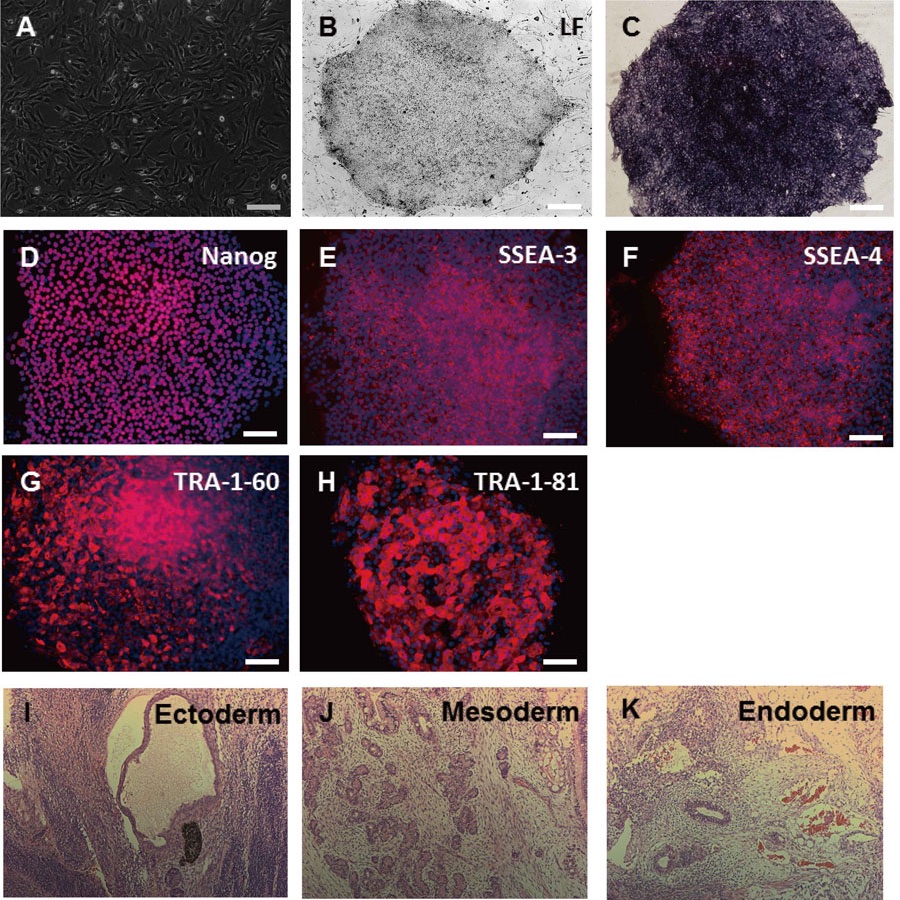
**

**Supplementary figure. S1 Identification of the generation of hiPS cells from HDF.**

(A) Morphology of human newborn foreskin fibroblasts; (B) Typical morphology of hES-like hiPS colony; (C) AP staining of the hiPS colony; (D-H) Immunocytochemistry for Nanog (D), SSEA-3 (E), SSEA-4 (F), TRA-1-60 (G), TRA-1-81 (H); (I-K) Nine weeks after transplanting human iPS cells subcutaneously into dorsal flanks of immunodeficient (SCID) mice, it showed the tumor formation. Histology of teratomas derived from iPS cells, ectoderm (I), mesoderm (J), endoderm (K). Nuclei were stained with DAPI (blue). Bars = 200 mm (A-H).

Supplementary Table. 1 The primer sequences used in qPCR

| Name | Sequences | |
| --- | --- | --- |
| β-Actin-hum | Sense: 5’ CCAGGGCGTTATGGTAGGCA 3’  Antisense: 5' TTCCATATCGTCCCAGTTGGT 3’ |  |
| pet-1-hum | Sence: 5’ TCAGAAAGGCAGCGGACAGAT 3'  Antisence: 5’ GGTCCGTGAGCTTGAACTCG 3’ |  |
| htr1a-hum | Sense: 5’ CTGCGCTCATCTCGCTCACTT 3’  Antisense: 5’ CAGCAGCGGGATGTAGAAAGC3’ |  |
| htr1b-hum | Sense: 5’ CACGGTGGGTGCTTTCTACTTC 3’  Antisense:5’GAGTTAATAGAGGTGACCGAGGACG3’ |  |
| htr1d-hum | Sense: 5’ TCAGCATCGCCTATACCATCACC 3’  Antisense: 5’ CCGTCCTGCGTTTACTGTATTCC 3’ |  |
| htr1f-hum | Sense: 5’ CAACTCCCTTGTGATCGCTGC 3’  Antisense: 5’ GCAGGTAATGTCAACACTCAGCC 3’ |  |
| htr2a-hum | Sense: 5’ GCCATCCAGAATCCCATCCAC 3’  Antisense: 5’ CAGGACAAAGTTATCATCGGCG 3’ |  |
| htr2b-hum | Sense: 5’ AAGCCACCTCAACGCCTAACAT 3’  Antisense: 5’ TGAGTTGGGCAGAGCCTTGTC 3’ |  |
| htr5a-hum | Sense: 5’ AGCCTTCCTACGCCGTGTTCT 3’  Antisense: 5’ CTGTTTGGCAGAGTCCTTCACC 3’ |  |
| htr1aa-zebr | Sense: 5’ TTCTACATCCCGCTCATCCTCA 3’  Antisense: 5’ CCTCCAAGTTTTACCCACCTCTC 3’ |  |
| htr1ab-zebr | Sense: 5’ AAACACCGAGGCGAAGAGGAA 3’  Antisense: 5’ GGCAGCCAACACAGAATGAAAGT 3’ |  |
| htr1b-zebr | Sense: 5’ GCTACGTCAACTCACTCATCAA 3’  Antisense: 5’ TCCTATCGTCTGCAACATCTAAA 3’ |  |
| htr2a-zebr | Sense: 5’ TACGGTGGCTGGGAACATTTTAG 3’  Antisense: 5’ GGGACACAGTGATGCAGGGAAA 3’ |  |
| htr5a-zebr | Sense: 5’ TGGATCAAAGAGGACCAACACC 3’  Antisense: 5’ CTGAAACGTCACCGTGGCAT 3‘ |  |

Supplementary Table. 2 The F-value and p-value for data analysis

| Figure.2 | | | |
| --- | --- | --- | --- |
|  | F-value | F-Distribution (alpha=0.05) * | p-value |
| pet-1 | 185.2 | 3.8379 | P < 0.0001 |
| htr1a | 134.6 | 3.8379 | P < 0.0001 |
| htr1b | 43.7 | 3.4780 | P < 0.0001 |
| htr1d | 2270 | 3.4780 | P < 0.0001 |
| htr1f | 27.52 | 3.8379 | P < 0.0001 |
| htr2a | 18.96 | 3.8379 | P = 0.0001 |
| htr2b | 66.96 | 3.8379 | P < 0.0001 |
| htr5a | 23.50 | 3.8379 | P < 0.0001 |

| Figure.3 | | | |
| --- | --- | --- | --- |
|  | F-value | F-Distribution (alpha=0.05) * | p-value |
| pet-1 | 214.4 | 5.1433 | P < 0.0001 |
| htr1a | 38.58 | 5.1433 | P < 0.0001 |
| htr1b | 143.8 | 5.1433 | P < 0.0001 |
| htr1d | 23.61 | 5.1433 | P =0.0014 |
| htr1f | 108.1 | 5.1433 | P < 0.0001 |
| htr2a | 4.189 | 5.1433 | P = 0.0728 |
| htr2b | 16.3 | 5.1433 | P =0.0038 |
| htr5a | 33.34 | 5.1433 | P =0.0006 |

Figure 4:

| HTR1aa | | | |
| --- | --- | --- | --- |
|  | F-value | F-Distribution (alpha=0.05) * | p-value |
| Time points factor | 34.84 | 2.313 | P < 0.0001 |
| Doses factor | 29.20 | 3.209 | P < 0.0001 |
| Time points x Doses (Interaction) | 10.87 | 1.97 | P < 0.0001 |

| HTR1ab | | | |
| --- | --- | --- | --- |
|  | F-value | F-Distribution (alpha=0.05) * | p-value |
| Time points factor | 17.67 | 2.336 | P < 0.0001 |
| Doses factor | 14.85 | 3.232 | P < 0.0001 |
| Time points x Doses (Interaction) | 1.096 | 2.000 | P = 0.3898 |

| HTR1b | | | |
| --- | --- | --- | --- |
|  | F-value | F-Distribution (alpha=0.05) * | p-value |
| Time points factor | 14.39 | 2.380 | P < 0.0001 |
| Doses factor | 9.615 | 3.276 | P = 0.0005 |
| Time points x Doses (Interaction) | 1.228 | 2.00 | P = 0.3051 |

| HTR2a | | | |
| --- | --- | --- | --- |
|  | F-value | F-Distribution (alpha=0.05) * | p-value |
| Time points factor | 19.96 | 2.33 | P < 0.0001 |
| Doses factor | 9.003 | 3.23 | P = 0.0006 |
| Time points x Doses (Interaction) | 1.061 | 1.700 | P =0.4158 |

| HTR5a | | | |
| --- | --- | --- | --- |
|  | F-value | F-Distribution (alpha=0.05) * | p-value |
| Time points factor | 31.61 | 2.014 | P < 0.0001 |
| Doses factor | 36.43 | 2.519 | P < 0.0001 |
| Time points x Doses (Interaction) | 2.196 | 1.809 | P =0.0454 |

| Figure. 7(B, E) | | | |
| --- | --- | --- | --- |
|  | F-value | F-Distribution (alpha=0.05) * | p-value |
| B (6dpf) | 617.3 | (3.01, 3.04) | P < 0.0001 |
| E (12dpf) | 413.2 | (3.01, 3.04) | P < 0.0001 |

*The F-Distribution are from F Table for α = 0.05. ^[3,4]^

Reference

1. Takahashi, K., et al. Induction of pluripotent stem cells from adult human fibroblasts by defined factors. *Cell* **131**, 861-872 (2007).

2. Park, I. H., Lerou, P. H., Zhao, R., Huo, H. & Daley, G. Q. Generation of human-induced pluripotent stem cells. *Nat. Protoc.* **3**, 1180-1186 (2008).

3. Abramowitz, M., & Stegun, I. A. Handbook of mathematical functions: with formulas, graphs, and mathematical tables (Vol. 55). (Courier Corporation, 1964)

4. Sematech, N. I. S. T. Engineering statistics handbook. (NIST SEMATECH, 2006).
